# Supplementary material for: Investigating leaf beetles (Coleoptera, Chrysomelidae) on the west coast islands of Sabah via checklist-taking and DNA barcoding
Source: PeerJ. 2018 Oct 25;6:e5811. doi: 10.7717/peerj.5811 (PMC6204241; doi:10.7717/peerj.5811)
Supplement: Supplemental Information 1 [file peerj-06-5811-s001.docx]

**Table 3: Top-hit search results from NCBI GenBank and Barcode of Life Data System (BOLD)**

| **Query ID** | **BOR/COL** | **GenBank** | | | **BOLD** | |
| --- | --- | --- | --- | --- | --- | --- |
|  |  | **Pairwise identity (%)** | **Species name** | **GenBank Accession** | **Pairwise identity (%)** | **Species name** |
| *Brontispa longissima* | 8054 | 100.00 | *Brontispa longissima* | JQ302136 | 100 | *Brontispa longissima* |
| *Brontispa longissima* | 8397 | 100.00 | *Brontispa longissima* | JQ302136 | 100 | *Brontispa longissima* |
| *Brontispa longissima* | 8453 | 100.00 | *Brontispa longissima* | JQ302136 | 100 | *Brontispa longissima* |
| *Altica aenea* | 8071 | 99.20 | *Altica birmanensis* | KX778628 | 100 | *Altica* sp. |
| *Altica aenea.* | 8166 | 99.00 | *Altica engstroemi* | KX778636 | 100 | *Altica* sp. |
| *Monolepta* sp. 7 | 8426 | 98.00 | Galerucinae sp. | KF946433 | 90.52 | Chrysomelidae sp. |
| *Hoplosaenidea variabilis* | 9638 | 97.90 | *Theopea* sp. | AB794770 | 97.84 | *Theopea sp.* |
| *Monolepta* sp. 17 | 9449 | 97.10 | *Monolepta* sp. | AB794728 | 97.04 | *Monolepta* sp. |
| *Monolepta* sp. 8 | 8314 | 96.20 | *Monolepta* sp. | AB794731 | 96.14 | *Monolepta* sp. |
| *Monolepta* sp. 15 | 8456 | 93.00 | *Monolepta* sp. | AB794741 | 93.02 | *Monolepta* sp. |
| *Colasposoma auripenne* | 9753 | 91.80 | *Colasposoma dauricum* | LN995410 | 91.9 | *Colasposoma* sp. |
| *Monolepta* sp. 3 | 6924 | 91.40 | *Monolepta longitarsoides* | KC185734 | 91.37 | *Monolepta longitarsoides* |
| *Monolepta* sp. 4 | 6921 | 90.90 | *Monolepta* sp. | AB794741 | 92.42 | Coleoptera sp. |
| *Monolepta* sp. 2 | 6931 | 90.40 | *Monolepta* sp. | AB794753 | 90.39 | *Monolepta* sp. |
| *Hoplosaenidea* sp. 2 | 8538 | 90.30 | *Hoplosaenidea subcostata* | KC255439 | 90.24 | *Hoplosaenidea subcostata* |
| *Ochralea nigripes* | 8356 | 90.30 | Galerucinae sp. | KF946261 | 94.47 | *Monolepta* sp. |
| *Monolepta* sp. 5 | 9734 | 90.20 | *Monolepta* sp. | AB794757 | 93.87 | Chrysomelidae sp. |
| *Clitena* sp. | 8399 | 90.00 | *Clitea fulva* | KC185760 | 89.86 | Chrysomelidae sp. |
| *Ochralea nigripes* | 8362 | 90.00 | Galerucinae sp. | KR425397 | 94.32 | *Monolepta* sp. |
| *Monolepta* sp. 5 | 8178 | 90.00 | *Monolepta* sp. | AB794757 | 93.52 | Chrysomelidae sp. |
| *Monolepta* sp. 5 | 8180 | 90.00 | *Monolepta* sp. | AB794753 | 93.6 | Chrysomelidae sp. |
| *Monolepta* sp. 5 | 8403 | 89.90 | *Monolepta* sp. | AB794757 | 93.9 | Chrysomelidae sp. |
| *Ochralea nigripes* | 8411 | 89.80 | Galerucinae sp. | KR425397 | 94.47 | *Monolepta* sp. |
| *Monolepta* sp. 18 | 9679 | 89.80 | *Monolepta longitarsoides* | KC185734 | 92.86 | Chrysomelidae sp. |
| *Monolepta* sp. 5 | 9718 | 89.80 | *Monolepta* sp. | AB794757 | 93.64 | Chrysomelidae sp. |
| *Ochralea nigripes* | 8015 | 89.70 | Galerucinae sp. | KR425397 | 94.16 | *Monolepta* sp. |
| *Monolepta* sp. 5 | 8348 | 89.70 | *Monolepta* sp. | AB794757 | 93.52 | Chrysomelidae sp. |
| *Monolepta* sp. 5 | 9719 | 89.70 | *Monolepta* sp. | AB794753 | 93.55 | Chrysomelidae sp. |
| *Aulacophora* sp. | 8103 | 89.70 | *Atrachya* sp. | KC185693 | 89.74 | *Atrachya* sp. BMNH 846510 |
| *Monolepta* sp. 5 | 9897 | 89.60 | *Monolepta* sp. | AB794753 | 93.39 | Chrysomelidae sp. |
| *Ochralea nigripes* | 8408 | 89.50 | Galerucinae sp. | KR425397 | 94.01 | *Monolepta* sp. |
| *Monolepta* sp. 11 | 8119 | 89.50 | *Monolepta longitarsoides* | KC185734 | 92.72 | Coleoptera sp. |
| *Dercetina* sp. | 8150 | 89.20 | *Atrachya* sp. | KC185693 | 89.71 | Coleoptera sp. |
| *Monolepta* sp. 10 | 8104 | 89.10 | Galerucinae sp. | KJ677806 | 90.75 | Coleoptera sp. |
| *Monolepta* sp. 10 | 8181 | 89.10 | Galerucinae sp. | KJ677806 | 90.75 | Coleoptera sp. |
| *Monolepta* sp. 9 | 9440 | 89.00 | *Monolepta longitarsoides* | KC185734 | 89.45 | Chrysomelidae sp. |
| *Monolepta* sp. 9 | 9825 | 89.00 | *Monolepta longitarsoides* | KC185734 | 89.71 | Chrysomelidae sp. |
| *Monolepta* sp. 9 | 8525 | 88.90 | Galerucinae sp. | KJ677802 | 89.74 | Chrysomelidae sp. |
| *Dercetina* sp. | 8428 | 88.90 | *Monolepta quadriguttata* | KC135967 | 89.76 | Coleoptera sp. |
| *Aphthona* sp. | 9602 | 88.60 | *Longitarsus candidulus* | KF654954 | 89.02 | Chrysomelidae sp. |
| genus indet. nr. *Monolepta* | 8277 | 88.10 | *Monolepta longitarsoides* | KC185734 | 100 | Chrysomelidae sp. |
| genus indet. nr. *Monolepta* | 9875 | 88.10 | *Monolepta longitarsoides* | KC185734 | 100 | Chrysomelidae sp. |
| genus indet. nr. *Monolepta* | 9893 | 88.10 | *Monolepta longitarsoides* | KC185734 | 100 | Chrysomelidae sp. |
| *Hoplosaenidea malayensis* | 8330 | 87.90 | *Hoplosaenidea subcostata* | KC255439 | 86.36 | Chrysomelidae sp. |
| *Erystus villicus* | 8134 | 87.80 | *Longitarsus tabidus* | KF654096 | 87.19 | Chrysomelidae sp. |
| *Hoplosaenidea malayensis* | 8425 | 87.80 | *Hoplosaenidea subcostata* | KC255439 | 86.36 | Chrysomelidae sp. |
| *Hoplosaenidea malayensis* | 8440 | 87.80 | *Hoplosaenidea subcostata* | KC255439 | 86.39 | Chrysomelidae sp. |
| *Cleorina malayana* | 9597 | 87.50 | Eumolpinae sp. | KF946194 | 87.52 | Chrysomelidae sp. |
| *Hyphasis* sp. | 8449 | 87.40 | *Monolepta* sp. | AB794736 | 88.33 | Coleoptera sp. |
| *Monolepta* sp. 6 | 8531 | 87.40 | *Monolepta quadriguttata* | KF966604 | 88.89 | *Trirhabda* sp. |
| *Plagiodera* sp. | 8514 | 87.20 | *Chrysomela vigintipunctata* | KU188452 | 87.31 | Coleoptera sp. |
| *Basilepta* sp. 2 | 6930 | 87.10 | Chrysomelidae sp. | KX781753 | 88.79 | Coleoptera sp. |
| *Metrioidea grandis* | 8417 | 87.10 | *Hapalaraea* sp. | KU875173 | 88.07 | Chrysomelidae sp. |
| *Pagria* sp. | 9479 | 87.10 | Eumolpinae sp. | KF946272 | 98.8 | *Pagria* sp. |
| *Basilepta* sp. 1 | 8202 | 86.90 | Eumolpinae sp. | KF946257 | 89.3 | Coleoptera sp. |
| *Monolepta* sp. 12 | 9201 | 86.50 | *Lochmaea crataegi* | KM447871 | 87.87 | Coleoptera sp. |
| *Hoplosaenidea* sp. 1 | 7000 | 86.40 | *Paleosepharia posticata* | KY195975 | 87.46 | Chrysomelidae sp. |
| *Metrioidea grandis* | 8094 | 86.40 | *Phaedon armoraciae* | KC255426 | 87.31 | Chrysomelidae sp. |
| *Argopistes* sp. 1 | 8442 | 86.30 | *Monolepta* sp. | AB794741 | 89.55 | Chrysomelidae sp. |
| *Lema* sp. | 9393 | 86.30 | *Lema daturaphila* | KR481201 | 95.08 | Coleoptera sp. |
| *Argopistes* sp. 2 | 9608 | 86.20 | *Longitarsus atricillus* | KF134547 | 87.1 | *Caeporis stigmula* |
| *Phola sedecimpustulata* | 9882 | 86.20 | Galerucinae sp. | KR425406 | 86.82 | Chrysomelidae sp. |
| *Notosacantha* sp. 1 | 8312 | 86.10 | Hispinae sp. | KR424810 | 85.34 | Coleoptera sp. |
| *Lanka* sp. | 8097 | 86.00 | *Orestia punctipennis* | KF654864 | 86.9 | Chrysomelidae sp. |
| *Sumatrasia* sp. | 6938 | 85.80 | *Longitarsus parvulus* | KX943391 | 85.93 | *Mimastra limbata* |
| *Basilepta* sp. 4 | 8064 | 85.50 | Eumolpinae sp. | KF946194 | 85.69 | *Xanthonia decemnotata* |
| *Strobiderus* sp. | 6995 | 85.50 | *Psylliodes chrysocephalus* | KF653250 | 85.87 | Chrysomelidae sp. |
| *Monolepta* sp. 14 | 9418 | 85.30 | *Mantura chrysanthemi* | KF653804 | 85.94 | Chrysomelidae sp. |
| *Monolepta* sp. 14 | 9556 | 85.30 | *Mantura chrysanthemi* | KF653804 | 86.17 | Chrysomelidae sp. |
| *Monolepta* sp. 14 | 9557 | 85.30 | *Mantura chrysanthemi* | KF654246 | 85.74 | Chrysomelidae sp. |
| *Nodina* sp. | 8418 | 85.20 | *Colasposoma dauricum* | LN995410 | 86.74 | Chrysomelidae sp. |
| *Monolepta* sp. 1 | 8323 | 85.10 | *Monolepta atrimarginata* | KC185733 | 85.26 | Chrysomelidae sp. |
| *Monolepta* sp. 1 | 8427 | 85.10 | *Monolepta atrimarginata* | KC185733 | 85.17 | Chrysomelidae sp. |
| *Basilepta* sp. 3 | 8379 | 84.80 | Eumolpinae sp. | KF946194 | 85.37 | Chrysomelidae sp. |
| *Schenklingia* sp. | 9429 | 84.80 | *Psylliodes cucullatus* | KR486778 | 88.12 | Chrysomelidae sp. |
| *Notosacantha* sp. 2 | 8540 | 84.70 | *Dicladispa armigera* | KY845676 | 87.12 | *Pingasa rhadamaria alterata* |
| *Nodina* sp. | 8447 | 84.60 | Eumolpinae sp. | KF946194 | 86.39 | Chrysomelidae sp. |
| *Nodina* sp. | 8197 | 84.40 | Eumolpinae sp. | KF946194 | 85.93 | Chrysomelidae sp. |
| *Nodina* sp. | 8398 | 84.40 | Eumolpinae sp. | KF946194 | 86.51 | Chrysomelidae sp. |
| *Colaspoides* sp. 1 | 9398 | 84.10 | Eumolpinae sp. | KJ677941 | 85.69 | Chrysomelidae sp. |
| *Gonophora* sp. | 8260 | 84.10 | *Agrius convolvuli* | LC049959 | 84.2 | *Semomesia croesus* |
| *Gonophora* sp. | 8344 | 84.10 | *Agrius convolvuli* | LC049959 | 84.2 | *Semomesia croesus* |
| *Gonophora* sp. | 8423 | 84.10 | *Agrius convolvuli* | LC049959 | 84.2 | *Semomesia croesus* |
| *Hoplosaenidea* sp. 5 | 9721 | 84.10 | Galerucinae sp. | KJ677800 | 84.68 | *Rhigognostis senilella* |
| *Hoplosaenidea* sp. 4 | 8095 | 84.00 | *Longitarsus luridus* | KF134571 | 85.47 | Coleoptera sp. |
| *Hoplosaenidea* sp. 5 | 9720 | 84.00 | Galerucinae sp. | KJ677800 | 84.87 | *Rhigognostis senilella* |
| *Nodina* sp. | 8179 | 84.00 | *Colydiinae* sp. | KU873303 | 85.59 | Chrysomelidae sp. |
| *Scelodonta granulosa* | 9531 | 84.00 | Chrysomelidae sp. | KM842629 | 99.31 | *Scelodonta* sp. |
| *Nodina* sp. | 8510 | 83.90 | *Colaspidea globosa* | KF653259 | 87.03 | *Nodina* sp. |
| *Gonophora* sp. | 8016 | 83.80 | *Epinotia nigricana* | KP253547 | 84.01 | *Rhigognostis senilella* |
| *Dactylispa* sp. 1 | 9777 | 83.40 | *Callisto basistrigella* | KM253781 | 84.07 | Chrysomelidae sp. |
| *Colaspoides* *tuberculata* | 9858 | 83.20 | Eumolpinae sp. | KR424893 | 84.54 | Chrysomelidae sp. |
| *Rhyparida* sp. 2 | 8262 | 83.20 | Eumolpinae sp. | KF946328 | 86.24 | Coleoptera sp. |
| *Rhyparida* sp. 1 | 8355 | 83.00 | Eumolpinae sp. | KF946450 | 86.09 | Coleoptera sp. |
| *Dactylispa* sp. 2 | 8305 | 82.90 | *Monolepta* sp. | AB794736 | 85.34 | Coleoptera sp. |
| *Rhyparida* sp. 1 | 8422 | 82.90 | Eumolpinae sp. | KF946450 | 85.93 | Coleoptera sp. |
| *Rhyparida* sp. 1 | 8508 | 82.90 | Eumolpinae sp. | KF946450 | 85.93 | Coleoptera sp. |
| *Rhyparida* sp. 1 | 8450 | 82.70 | Eumolpinae sp. | KF946450 | 85.78 | Coleoptera sp. |
| *Rhyparida* sp. 1 | 8470 | 82.70 | Eumolpinae sp. | KF946450 | 85.71 | Coleoptera sp. |
| *Rhyparida* sp. 1 | 6939 | 82.60 | Eumolpinae sp. | KF946450 | 85.78 | Coleoptera sp. |
| *Rhyparida* sp. 1 | 8063 | 82.60 | Eumolpinae sp. | KF946450 | 85.78 | Coleoptera sp. |
