# Supplementary material for: Investigating leaf beetles (Coleoptera, Chrysomelidae) on the west coast islands of Sabah via checklist-taking and DNA barcoding
Source: PeerJ. 2018 Oct 25;6:e5811. doi: 10.7717/peerj.5811 (PMC6204241; doi:10.7717/peerj.5811)
Supplement: Supplemental Information 5 [file peerj-06-5811-s005.docx]

**Appendix A**

**Generic diagnosis of genera**

**Genus *Altica* Geoffroy, 1762.**

Gressitt & Kimoto (1963) provided the diagnosis key to the genus as following:

“Pronotum and elytra not densely pubescent. Pronotum with ante-basal transverse impression extending to the sides, not limited on sides by a short, longitudinal impression. Elytron with punctation irregular, confused or obsolete. Anterior coxal cavities open behind. Mesosternum not excavated in center. Hind femur not with a long, straight, apical spine, exceeding tibia in length. Hind tibia with apical spine simple or absent. Claw-segment of hind tarsus not strongly dilated.”

Reid & Beatson (2015) also provided a detailed generic diagnosis description of genus *Altica* based on Australian, Indomalayan and Pacific *Altica* species.

**Genus *Aphthona* Chevrolat, 1837.**

Gressitt & Kimoto (1963) provided the diagnosis key to this genus as following:

“Pronotum and elytra not densely pubescent. Antenna not with length of segment 1 attaining or exceeding the combined lengths of 2-4. Interantennal space usually narrow, with breadth much less than transverse diameter of eye. Postantennal tubercles prominent and subovate, not extending to interantennal space. Pronotum and elytra not densely pubescent. Pronotum evenly convex, without a distinct ante-basal, transverse impression. Elytron with punctation irregular, confused or obsolete. Anterior coxal cavities open behind. Prosternum exceeding posterior margin of coxae. metasternum not produced anteriorly, not concealing mesosternum. Hind femur not with a long, straight, apical spine, exceeding tibia in length. . Hind tibia with apical spine inserted laterally on apex, without, or with a short, subapical excavation. Hind tarsus with segment 1 distinctly shorter than 1/2 length of tibia. Tarsus with segment 3 bilobed. Claw-segment of hind tarsus not strongly dilated.”

**Genus *Argopistes* Motschulsky, 1860.**

Blanco & Konstantinov (2013) provided the generic diagnosis on genus *Argopistes* as following:

“*Argopistes* is easily recognized among world flea beetles based on a round body, an opistognathous head without the anterofrontal ridge with a very long and flat frons having a thin, sharp (along the edge) frontal ridge, plane of frons forming acute angle with respect to plane of vertex in lateral view, clypeus membrane-like, labrum transverse, 11 antennomeres, open procoxal cavities, greatly enlarged metafemora and a short, curved in lateral view, metatibia with the metatarsus and spur attached before the apex. Male *Argopistes* can be differentiated from females, as many other flea beetle genera, based on a relatively wide first protarsomere, which is much narrower in females.”

**Genus *Erystus* Jacoby, 1885.**

Maulik (1926) provided the generic diagnosis of this genus as following:

“Body broadly ovate. Head: eyes entire; antennae rather short and robust, the segments, with the exception of the first and the last, being almost equal in length. Prothorax narrowly transverse, sides strongly rounded, surface distinctly punctate. Scutellum small, broader than long, triangular. Elytra semi-depressed and dilated at the sides, regularly punctate-striate, the interstices somewhat costate, lateral margins broadened, their epipleura very broad, concave when seen from the ventral side. Underside: anterior coxal cavities closed; prosternum rather broad, obsoletely carinate; posterior femora strongly incrassate; tibiae short, dilated at the apex, the four anterior tibiae unarmed, the posterior pair with a small spine (in the genotype, but this spine is absent in *E. andamanensis*); their dorsal surface obsoletely channelled; tarsi broad and short, nearly equal in length; claws appendiculate.”

**Genus *Hemipyxis* Chevrolat, 1836.**

Gressitt & Kimoto (1963) provided the diagnostic key to this genus as following:

“Body usually ovate (=*Sebaethe*). Interantennal space usually narrow, with breadth much less than transverse diameter of eye. Postantennal tubercles subquadrate or transverse. Interantennal space carinate. Length of antenna segment 1 not attaining or exceeding the combined lengths of 2-4. Pronotum and elytra not densely pubescent Pronotum evenly convex, without a distinct ante-basal, transverse impression. Anterior coxal cavities open behind. Prosternum exceeding posterior margin of coxae; metasternum produced anteriorly, concealing mesosternum. Elytron with punctation irregular, confused or obsolete. Hind femur not with a long, straight, apical spine, exceeding tibia in length. Hind tibia with apical spine simple or absent, with an axial excavation extending from apex to basal 1/4 or more. Tarsus with segment 3 bilobed. Claw-segment of hind tarsus not strongly dilated.”

**Genus *Hyphasis* Harold, 1877**

Maulik (1926) provided the diagnosis description to this genus as following:

“Body oblong-ovate. Head broader than long, vertex convex, narrow, interocular space narrow, frontal tubercles in the interantennal space well developed and with a longitudinal impression along the middle. Antennae passing a little beyond the middle of the elytra, slender; first segment elongate, club-shaped, second very small, less than half the length of the first or the third; from the third to the seventh the segments are more or less nearly equal to each other in length, from the eighth to the eleventh they are shorter, the last being pointed; the whole antennae are covered with pubescence. Eyes strongly convex. Prothorax much broader than long, upper surface somewhat depressed, the lateral margins somewhat explanate, the explanate portion being concave; anterior margin nearly straight, posterior slightly sinuate, anterior and posterior lateral angles rounded. Scutellum moderately large, triangular, with apex rounded. Elytra hardly broader at the base than the prothorax, the sides rather nearly parallel, with the margins slightly explanate and apex broadly rounded; surface closely, strongly and confusedly punctate. Underside: elytral epipleura extraordinarily broad; posterior femora with a channel on the underside; posterior tibiae with a pointed spine at the apex; claw-segment of the posterior tarsi swollen; claws simple.”

**Genus *Lanka* Maulik, 1926.**

Maulik (1926) provided the original description of this genus as following:

“Body oblong-ovate, small. Head as broad as prothorax; eyes moderately convex; vertex convex and continuing as a fine ridge between the bases of the antennae. Antennae only about a millimetre shorter than the body, with apical segments slightly thickened. Prothorax quadrate, convex, and sloping down steeply on each side in front, base widely rounded, sides margined, anterior and posterior angles rounded; no ante-basal furrow. Scutellum small, triangular, with apex rounded. Elytra only slightly broader at base than prothorax, regularly punctate-striate, the punctures being extremely fine and placed rather far apart from each other in each row. The whole of the upper surface is otherwise perfectly smooth. Underside: anterior coxal cavities open behind; prosternum moderately broad and rounded at apex; metasternum prominently elevated, each side being formed into a lobe with a rounded extremity; epipleura of elytra continuing almost up to the apex; posterior femora strongly incrassate; tibiae subcylindrical, not channelled; the posterior pair have the upper side more or less flattened and a sharp, small, apical spine; first segment of anterior and middle tarsi somewhat broadened (this may be a secondary sexual character), that of the posterior tarsi not broadened, but almost equal in length to the two following segments; claws appendiculate.”

**Genus *Schenklingia* Csiki & Heikertinger, 1940**

Samuelson (1969) provided the diagnosis description for this genus as following:

“Frons triangular, antennal insertions very close; antennal scape long, as long as segments 2+3 or 2+3+4 together; pronotum lacking antebasal impression, base sinuate; elytral punctures in longitudinal rows, sometimes with internal rows partly irregular; procoxal cavities open behind; mesosternum concealed; metatibia channeled; claws appendiculate.”

**Genus Aulacophora Dejean, 1835**

Gressitt & Kimoto (1963) provided diagnostic key to this genus as following:

“Antennal insertions generally separated, situated near, but behind anterior borders of eyes, but when weakly separated or placed farther forward, occiput and pronotum not heavily punctured. Pronotum with a transverse depression, sometimes divided at middle. Elytral epipleuron abbreviated behind middle and strongly narrowed behind basal 1/3. Anterior coxal cavities open behind. Mid coxae contiguous or close, with space between them much less than width of a coxa. Prosternum weak or lacking between coxae. Mesosternum free, horizontal or inclined, not covered by metasternum. Last abdominal sternite of male 3-lobed, with median lobe always distinct. Tibiae distinctly spined apically. Tarsal claws bifid.”

**Genus *Strobiderus* Jacoby, 1884**

Jacoby (1884) provided the original description of this genus as following:

“Body oblong. Head exserted. Eyes large entire. Palpi robust, last joint conical. Antennae slender, filiform, long, pubescent, first joint very long, second one short, third joint scarcely shorter than the first, the rest elongate equal. Thorax transverse, convex, without depression. Elytra deeply punctate-striate, interstices longitudinally costate, pubescent, the apex produced into a flattened protuberance and preceded by a deep inward excavation (male). Prosternum not visible, anterior coxal cavities closed. Legs slender, elongate posterior tibiae with spine, their first tarsal joint of half their length. Claws appendiculate. Female apex of the elytra simple.”

**Genus *Hoplosaenidea* Laboissiere, 1933.**

Laboissiere (1933) provided the original description in French and Kimoto (1989) provided the diagnostic key to this genus as following:

“Head with frontal tubercles not widely separated by frons, anterior margin of clypeus entire. Antenna1 insertions generally separated, situated near, but behind anterior margins of eyes. When separated or placed further forward, vertex and pronotum not heavily punctate. Pronotum with basal margin entirely marginate and a pair of distinct depressions laterally, without longitudinal furrows; distinctly marginate on lateral borders. Elytral punctures not regularly striated. Mesosternum free, horizontal or inclined, not covered by a process of metasternum. Anterior coxal cavity closed posteriorly. Last abdominal segment of male trilobed, with median lobe always distinct. Posterior tibia with a single spine at apex. First segment of posterior tarsus usually shorter than, or sometimes subequal to, remainder combined. Tarsal claws not bifid.”

**Genus *Metrioidea* Fairmaire, 1881.**

Fairmaire (1881) provided the original description in French and Mohamedsaid (1994) provided the diagnosis description as following:

“Head impunctate. Pronotum strongly convex; borders margined, anterior coxal cavity closed behind. Elytra sparsely pubescent, the epipleuron broad, extended to apex. In male, apical sternite trilobed. First segment of hind tarsus shorter than segments 2 and 3 combined. Tarsal claws appendiculate.”

**Genus *Monolepta* Erichson, 1843**

Maulik (1936) provided a comprehensive diagnosis description for this genus and Kimoto (1989) provided the diagnosis key to this genus as following:

“Antenna1 insertions generally separated, situated near, but behind anterior margins of eyes. When separated or placed further forward, vertex and pronotum not heavily punctate. Anterior coxal cavity closed posteriorly. Pronotum distinctly marginate on lateral borders. Elytral epipleuron suddenly narrowed at end of basal l/3 and distinctly narrower at middle than l/2 width in basal portion. Mesosternum free, horizontal or inclined, not covered by a process of metasternum. Last abdominal segment of male trilobed, with median lobe always distinct. Tibia with a long spine at apex. First segment of posterior tarsus much longer than remainder combined. Tarsal claws not bifid.”

**Genus *Ochralea* Clark, 1865**

Hazmi & Wagner (2010) redescribed this genus and provided diagnosis of this genus as following:

“7.75 – 14.40 mm body length with elongate basal metatarsomeres; dorsal colouration entirely yellow to yellowish- or brownish-red; second and third antennomeres short and almost the same size (ratio of the second to third antennomere 0.67 – 0.90 mm); pronotum comparatively narrow (length to width ratio of 0.62 – 0.73); procoxal cavitites partly open; median lobe deeply incised apically; tectum not incised; endophallic sac clearly visible with endophallic structures consisting of pairs of strong spiculae.”

**Genus *Clitena* Baly, 1864.**

Maulik (1936) provided the original description of this genus and Gressitt & Kimoto (1963) provided diagnostic key to this genus as following:

“Head large, not deeply sunken into prothorax. Occiput with a longitudinal groove. Occiput and pronotum deeply punctured. Antennal insertions generally close, at level of anterior margins of eyes or farther anterior. Gena shorter than eye. Labrum with irregular setigerous pores on each side. Pronotum more or less concave; raised on anterior border; with separate lateral depressions, and median portion raised and often grooved; somewhat rounded or sinuate on complete lateral margin, with primary setigerous pore on anterior corner. Anterior coxal cavities open or partly open behind. Mid coxae contiguous or close, with space between them much less than width of a coxa. Prosternum weak or lacking between coxae. Mesosternum free, horizontal or inclined, not covered by metasternum. Pronotum and elytra thickly or sparsely covered by hairs. Elytral epipleuron distinct at least in basal 1/2. Subbasal area of elytron not separated from behind by transverse depression. Last abdominal sternite of male with a triangular or rounded depression with posterior border often emarginate, but never 3-lobed. Tarsal claws bifid.”

**Genus *Sumatrasia* Jacoby, 1884**

Jacoby (1884) provided the original description of this genus as following:

“Body elongate, glabrous above. Head transversely grooved; frontal tubercles and carina distinct. Eyes regularly rounded, entire. Palpi robust, third and fourth joints of equal length, the latter conical. Antennae filiform, second joint one half the size of the third, the latter and following joints nearly equal. Thorax subquadrate, slightly broader than long, convex, narrowed anteriorly and posteriorly, anterior angles obsolete, posterior ones acute. Scutellum broad, subtrigonate, its sides rounded. Elytra elongate, punctate-striate, their epipleurae continued to the sutural angle. Legs slender. Tibiae unarmed, posterior first tarsal joint slightly longer than the 2 following ones united. Claws appendiculate. Prosternum extremely narrow. Anterior coxal cavities open.”

**Genus *Dercetina* Gressitt & Kimoto, 1963.**

Clark (1865) provided original description of this genus in Latin and Gressitt & Kimoto (1963) provided the diagnostic key to genus *Dercetina* as following:

“Eye rather large; Gena distinctly narrower than 1/3 of transverse diameter of an eye. Antennal insertions generally separated, situated near, but behind anterior borders of eyes, but when weakly separated or placed farther forward, occiput and pronotum not heavily punctured. In male antennal segment 3 distinctly longer than 2; 4 distinctly shorter than 2 + 3. Pronotum not very short, with or without a pair of depressions, without longitudinal furrows; both anterior and basal borders distinctly margined. Mesosternum free, horizontal or inclined, not covered by metasternum. Anterior coxal cavities closed behind. Mid coxae contiguous or close, with space between them much less than width of a coxa; prosternum weak or lacking between coxae. Elytron without rugosities. Last abdominal sternite of male 3-lobed, with median lobe always distinct. First segment of posterior tarsus usually shorter than, or sometimes subequal to, remainder combined. Posterior tibia with many short spines at apex. Tarsal claws appendiculate or simple.”

**Genus *Scelodonta* Westwood, 1837.**

Jacoby (1908) provided the diagnosis description of this genus as following:

“Body short, oblong; head with deep supraocular sulci; antennae short and robust, antennae with basal joint thickened, second very short, terminal joints short and thick. Thorax short, subglobular, much narrower than the elytra; lateral margins obsolete. Elytra short. Legs robust, femora dentate; tibiae entire or very slightly emarginate at apex; claws bifid. Prosternum transverse; thoracic episternum concave. The species are more or less metallic and pubescent; the thorax is frequently transversely strigose; elytra generally with rows of punctures and fine hairs, the interstices more or less finely wrinkled.”

**Genus *Colasposoma* Laporte, 1833.**

Baly (1865) provided the diagnosis description of this genus in Latin and Kimoto & Gressitt (1982) provided the diagnosis key to this genus as following:

“Body above glabrous. Head without sulcus above eye. Anterior margin of proepisterna straight or concave. Claws bifid.”

**Genus *Aulacia* Baly, 1867.**

Baly (1865) provided the original description in Latin and Medvedev (2009) provided the diagnosis key to this genus as following:

“Body ovate and small (2.5–5.0 mm). Colour not metallic and upperside not pubescent. Head with deeply sulcate groove bordered upper and inner margin of eye. Antennal segment 3 usually as long as 4 or shorter. Antennae without widened apical segments. Mandibles of male not prominent. Prothorax distinctly transverse, as broad as elytra at base, with lateral margins visible from above. Prosternum not produced anteriorly, rarely with collar bent downwards. Anterior margin of propleura distinctly convex, anterior margin of prosternum without collar. Elytra confusedly punctate, but with short rows along suture and apically. Elytra distinctly narrowed posteriorly, with acute apices and mostly with sharp lateral ridges, especially in females. Furrow of pygidium sharply delimited laterally. Spermatheca elongate, mostly arcuate or curved C- or U-like. Ductus of spermatheca not spiraled or often with simple spirale. Male: hind femora often with acute tooth, but never with finger-like projection; hind tibiae not emarginate on innerside.”

**Genus *Colaspoides* Laporte, 1833.**

Baly (1865) provided the diagnosis description of this genus in Latin and Medvedev (2009) provided the diagnosis key to this genus as following:

“Body ovate and mostly of moderate size, glabrous on dorsum. Colour variable. Head without deeply sulcate groove bordered upper and inner margin of eye. Antennae without widened apical segments, with segment 3 usually as long as 4 or shorter. Mandibles of male not prominent. Prothorax distinctly transverse, broad as elytra at the base, with lateral margins visible from above. Elytra confusedly punctate, but with short rows along suture and apically; mostly feebly narrowed posteriorly, with broadly rounded apices, but without sharp lateral ridges. Prosternum not produced anteriorly, rarely with collar bent downwards. Anterior margin of propleura distinctly convex, anterior margin of prosternum without collar. Furrow of pygidium sharply delimited laterally. Spermatheca elongate, mostly arcuate or curved C- or U-like, ductus often with simple spirale. Male: hind femora often with acute tooth, but never with finger-like projection; hind tibiae not emarginate on innerside.”

**Genus *Basilepta* Baly, 1860**

Baly (1860) provided the original description of this genus in Latin and Kimoto & Gressitt (1982) provided the diagnosis key to this genus as following:

“Body above glabrous. Head without sulcus above eye. Antenna slender, filiform, long, and often more than 2/3 as long as body length. Prothorax narrower than elytron at base. Anterior margin of proepisterna straight or concave. Intermediate and posterior tibiae, or intermediate tibia emarginate at apex. Claws appendiculate or simple.”

**Genus *Nodina* Motschulsky, 1858.**

Baly (1865) provided the diagnosis description of this genus in Latin and Kimoto & Gressitt (1982) provided the diagnosis key to this genus as following:

“Body generally very small and rounded, glabrous above. Head without sulcus above eye. Antenna filiform, robust and nearly as long as in preapical segments, and usually less than l/2 as long as body length. Prothorax as broad as elytron. Anterior margin of proepisterna straight or concave. Intermediate and posterior tibiae, or intermdiate tibia emarginate at apex. Claws appendiculate or simple.”

**Genus *Pagria* Lefevre, 1884.**

Lefevre (1884) provided the original description of this genus in Latin and Kimoto & Gressitt (1982) provided the diagnosis key to this genus as following:

“Body above glabrous. Head with sulcus above eye. Antenna filiform. Anterior margin of proepisterna straight or concave. Intermediate and posterior tibiae, or intermediate tibia emarginate at apex. Claws appendicualte or simple.”

**Genus *Rhyparida* Baly, 1861.**

Baly (1860) provided the original description of this genus in Latin and Gressitt (1969) provided the diagnosis key to this genus as following:

“Body broad as a rule. Dorsum fairly smooth, not coarsely vermiculate-punctate. Head generally with facial suture in form of inverted "Y"; with a shallow groove above eye (groove rarely deep). Occiput not greatly swollen. Interocular space often narrower than anterior margin of frontoclypeus. Neck not long. Prothorax broader than long, with distinct lateral margin. Anterior setigerous pore of prothorax near anterior end of lateral margin. Elytral apex not produced downward. Fore and hind femora both lacking a tooth beneath, or with minute teeth. Claws bifid, or semibifid and briefly appendiculate.”

**Genus *Cleorina* Lefevre, 1885.**

Jacoby (1908) provided the diagnosis description of this genus as following:

“Body very round and convex, generally highly metallic. Head deeply inserted; eyes rather large, oblong, feebly emarginated on their inner orbits; clypeus not separated from the face. Antennae filiform, short, third joint scarcely twice as long as the second, terminal joints widened. Thorax transverse, convex, narrower at base and apex, deeply transversely sulcate near the anterior margin, sides often straight. Elytra wider at base than the thorax, more or less strongly excavated below the base, punctate-striate, near the lateral margins longitudinally sulcate; epipleurae rounded, prominent. Legs moderately robust; femora unarmed; claws appendiculate. Prosternum very broad, flat, the base truncate. Anterior margin o£ thoracic episternum convex.”

**Genus *Brontispa* Sharp, 1904.**

Sharp (1904) provided the original description of this genus in Latin with following notes in English:

“The simply acuminate spinose projection on the front of the head, and the remarkably even surface (very regularly punctured) of the extremely elongate elytra, are sufficient to distinguish it. It has another remarkable character on the under surface of the head, the parts of the mouth are not closely applied to the front of the prosternum, but are separated from it by a curved ridge running all across the head in correspondence with the curve of the front of the prosternum.”

**Genus *Gonophora* Baly, 1858.**

Maulik (1919) provided the diagnosis description and key to this genus as following:

“Anterior margin of the mouth cavity not close to the base of the antennae. Prothorax not cylindrical, broadest in the middle, the lateral margins as a rule toothed and rough. Sides of the prothorax not parallel; the costae on the elytra much higher and well-developed throughout, never broadened and flattened at base.”

**Genus *Dactylispa* Weise, 1897.**

Maulik (1919) provided the comprehensive diagnosis description of this genus and Gressitt & Kimoto (1963) provided the diagnosis key to the genus as following:

“Body armed with spines on dorsal surfaces and lateral margins; form generally somewhat flattened and more or less oblong (Hispini). A spine, or group of spines, on each side of middle of anterior margin of pronotum. Antenna lacking spines entirely.”

**Genus *Notosacantha* Chevolat, 1836.**

Borowiec, Takizawa & Swietojańska (2013) provided the generic diagnosis of this genus as following:

“Small to moderate cassids with body length 2–8 mm. Body shape varies from elongate, parallel sided to circular. Pronotum more or less semicircular, its anterior margin usually deeply emarginate above head, thus head visible from above. Lateral margins of pronotum usually serrate, or spinulose, rarely simple, basal corners usually distinct, occasionally sides of pronotum broadly rounded then basal corners indistinct. Disc moderately convex, usually with shallow transverse impression behind anterior emargination, more or less marked transverse impression at base, and oblique short impression on each side of disc. Impressions usually punctate, top of disc in most species impunctate. Explanate margin of pronotum with large pores arranged irregularly, along sides of disc usually runs regular row of small pores. Scutellum large, usually triangular. Base of elytra in most species not or only slightly wider than base of pronotum, sides of elytra straight or convex but occasionally more or less emarginate in the middle. Disc of elytra with characteristic costae and tubercles forming homological structures, only few species have elytral sculpture forming only row of punctures without tubercles or costae. Explanate margin of elytra usually broad, more or less horizontal, often forming a gutter, surface with large, irregular pores or impunctate, transparent areas, especially below humeri. Elytral margin often serrate or spinulose. Head orthognathous, clypeus horizontal, often with more or less developed impressions, with or without median, longitudinal carina. Frons often forms a more or less flattened plate. Prosternal collar short, mouthparts visible, prosternal process moderately broad, strongly expanded apically. Antennal insertions adjacent, situated medially, antennae short, apical 4–5 segments form more or less distinct club. Legs stout, femora and tibiae without spines or teeth, tarsi very broad, with densely haired soles, last segment short, not extending beyond anterior margin of sole, claws simple.”

**Genus *Plagiodera* Chevrolat, 1837.**

Kimoto & Gressitt (1981) provided the diagnosis key to this genus as following:

“Body convex. Margination of anterior margin of pronotum entire. Base of pronotum margined. Round elytron with punctures confused or in irregular rows and without a costa. Elytral epipleuron concave, with outer border sharp and interior border not ciliate. Anterior coxal cavities open posteriorly. Mesosternum shorter than prosternum between coxae. Tibial apices not armed with toothlike processes. Tarsal segment tri-lobed. Tarsal claws simple.

**Genus *Phola* Weise, 1890.**

Kimoto & Gressitt (1981) provided the diagnosis key to this genus as following:

“Antenna subfiliform. Clypeus trapezoidal, not depressed. Anterior coxal cavities closed posteriorly. Tarsal claws appendiculate.”

**Genus *Lema* Fabricius, 1798.**

Kimoto & Gressitt (1979) provided the diagnosis key to this genus as following:

“Occiput not abbreviated, not wider than long, with sides forming a front angle of less than 90°. Prothorax generally broader than long, or as broad as long, side rather strongly constricted at or behind middle; elytron rarely much more than 3x as long as broad. Tarsal claws fused for basal ¼ to basal ½.”
